# The Role of CT-Based Attenuation Correction and Collimator Blurring Correction in Striatal Spect Quantification

**DOI:** 10.1155/2011/195037

**Published:** 2011-04-06

**Authors:** J. M. Warwick, S. Rubow, M. du Toit, E. Beetge, P. Carey, P. Dupont

**Affiliations:** ^1^Nuclear Medicine, Faculty of Health Sciences, Stellenbosch University and Tygerberg Hospital, Tygerberg, 7505, Cape Town, South Africa; ^2^MRC Unit for Stress and Anxiety Disorders, Faculty of Health Sciences, Stellenbosch University, Cape Town, South Africa; ^3^Laboratory for Cognitive Neurology and Medical Imaging Research Center, KU 3000 Leuven, Belgium

## Abstract

*Purpose*. Striatal single photon emission computed tomography (SPECT) imaging of the dopaminergic system is becoming increasingly used for clinical and research studies. The question about the value of nonuniform attenuation correction has become more relevant with the increasing availability of hybrid SPECT-CT scanners. In this study, the value of nonuniform attenuation correction and correction for collimator blurring were determined using both phantom data and patient data. *Methods*. SPECT imaging was performed using 7 anthropomorphic phantom measurements, and 14 patient studies using [I-123]-FP-CIT (DATSCAN). SPECT reconstruction was performed using uniform and nonuniform attenuation correction and collimator blurring corrections. Recovery values (phantom data) or average-specific uptake ratios (patient data) for the different reconstructions were compared at similar noise levels. *Results*. For the phantom data, improved recovery was found with nonuniform attenuation correction and collimator blurring corrections, with further improvement when performed together. However, for patient data the highest average specific uptake ratio was obtained using collimator blurring correction without nonuniform attenuation correction, probably due to subtle SPECT-CT misregistration. *Conclusions*. This study suggests that an optimal brain SPECT reconstruction (in terms of the lowest bias) in patients would include a correction for collimator blurring and uniform attenuation correction.

## 1. Introduction

Striatal single photon emission computed tomography (SPECT) imaging of the dopaminergic system is becoming increasingly used for clinical and research studies. Depending on the radiopharmaceutical, striatal uptake of activity may be a measure of binding to presynaptic receptors, for example, dopamine transporters (DAT), or postsynaptic receptors belonging to various neurotransmitter systems and/or subtypes thereof. The accurate quantification of striatal uptake with these studies is frequently important for their utilization in the clinical and research context. Accurate quantification of striatal SPECT imaging needs to take into account a number of factors that degrade image quality. These include photon attenuation, collimator blurring, photon scatter, and the limited spatial resolution of SPECT systems resulting in decreased count densities via partial volume effects [1].

Attenuation correction (AC) is implemented to correct for lower counts obtained from deeper brain structures. An early study using phantom data showed the value of iterative reconstruction incorporating corrections for attenuation and collimator blurring [2]. A simulation study showed that without any correction striatal recovery is underestimated by about 90%, improving to about 50% with attenuation correction (AC) and correction for scatter [3].

Uniform attenuation correction has a well-established role in brain SPECT studies [3–9]. Nonuniform attenuation correction has been found to result in a modest improvement in the quantification of regional cerebral blood flow (rCBF) studies [10–12]. The possible use of nonuniform attenuation correction has also become more relevant in recent years in light of the increasing availability of hybrid SPECT-computed tomography (CT) scanners. 

Arguably the most robust and commonly used uniform attenuation correction technique is that described by Chang [13]. This technique is based on two assumptions: (1) the scalp contour can be easily defined, (2) attenuation is constant throughout the head. It can be argued that while both of these assumptions have limitations in the context of brain SPECT imaging, radiopharmaceuticals concentrating predominantly in the basal ganglia (as opposed to the cortex) may result in SPECT images that are less reliable for the accurate definition of the scalp contour. 

In a study by Koch et al. [14] using uniform attenuation correction, the mean percentage difference between the measured and the predicted specific ratio (i.e., the ratio of activity in a striatal volume of interest (VOI) and an occipital background VOI) varied from 6.5 to 9.3% for different gamma camera/collimator combinations. It is possible that a significant proportion of this variability may have been due to difficulty in accurately defining the outline of the scalp from these images. While it is accepted that attenuation correction is required for striatal SPECT imaging, the value of nonuniform as opposed to uniform attenuation correction remains an issue requiring further clarification.

The finite length and width of collimator holes results in blurring of SPECT images. Software is commercially available for the reconstruction of SPECT images which correct for collimator blurring. Algorithms can allow for a fixed point spread function (PSF), or for the incorporation of a PSF which varies with distance from the collimator. Previous modeling studies [6, 7, 15] have demonstrated an improvement in signal recovery of 8–23% using these corrections. Some patient data has also been published showing the superiority of iterative reconstruction incorporating correction for collimator blurring over filtered backprojection [16, 17]. 

Another important source of image degradation is photon scatter, which can be corrected for by using a number of approaches [1, 15, 18, 19]. Scatter correction is included in simulations of striatal imaging in combination with other techniques [3, 15], independently contributing to a 12% improvement in recovery when using a parallel hole collimator [15]. Correction for partial volume effects have been found to be particularly important in improving recovery values in simulation studies [3, 15]. This has also been observed in a patient study [20]. An important challenge in techniques for correction of partial volume effects, however, is the requirement for a coregistered high-resolution anatomical image such as a Magnetic Resonance Imaging study. Corrections for photon scatter and partial volume effects will not however be studied in this paper.

In this study, the value for striatal SPECT imaging of (1) nonuniform attenuation correction using a low-dose CT (on a hybrid SPECT-CT scanner), and (2) constant and depth dependent corrections for collimator blurring, will be determined using data acquired both using an anthropomorphic striatal phantom, and from patient studies.

## 2. Methods

### 2.1. Phantom Measurements

All phantom measurements were performed using an anthropomorphic basal ganglia phantom commercially available from Radiology Support Devices Inc. (Long Beach, CA, USA). The study was performed using 7 phantom SPECT-CT studies, using 14 different specific uptake ratios (SUR) of Iodine-123, based on the following formula:

(1) $$\text{SUR}=\frac{\text{striatal}\;\text{activity}-\text{background}\;\text{activity}}{\text{background}\;\text{activity}}.$$

The study was performed using a methodology previously described [14]. All solutions were prepared in identical vials before the initial phantom SPECT acquisition. To minimise adhesion of I-123 to the plastic chamber walls, a 0.1 M sodium iodide solution was used as the basis for the mixtures. The striatal chambers (caudate and putamen chamber) on each side were filled with an identical activity concentration, with different activity concentrations on the left and right sides for each acquisition. For each successive acquisition, the striatal chambers were emptied, flushed, and then refilled with the next pair of concentrations. The large chamber simulating nonspecific background activity in the remainder of the brain was refilled for each acquisition with a similar activity of 5 kBq/mL, thus remaining similar during the whole experiment. SUR values were varied over a range approximating that seen in clinical practice.

To accurately determine the true SUR values achieved in the phantom, two separate 400 *μ*L samples were taken from each of the chambers directly after the SPECT measurements, and the activity concentrations of the samples were measured in a well counter (energy window 140–220 keV, counting time 10 minutes). The average counts/volume of each of the two identical samples was used to determine the true SUR values, using formula (1).

### 2.2. SPECT-CT Imaging

Data were acquired using a GE Hawkeye SPECT-CT gamma camera (GE Medical Systems, USA), equipped with low-energy high-resolution parallel hole collimators. The gamma camera operation and quality control conformed to that recommended by the camera manufacturers.

SPECT projection data were acquired with a 128 × 128 matrix with a zoom factor of 1.5, giving a pixel size of 2.95 mm. A circular orbit with a rotational radius of 15 cm (to collimator surface) was chosen for all phantom acquisitions. Acquisition was performed in step and shoot mode with both heads going through 120 steps each separated by 3 degrees. Each projection was acquired for 25 seconds, giving a total SPECT imaging time of about 50 minutes. Data were acquired at 159 keV with a 15% window. The SPECT acquisition was immediately followed by a low-dose CT study. This acquisition was done in accordance with the new guidelines of the EANM [21].

### 2.3. Reconstruction

All SPECT data were reconstructed with an ordered subsets expectation maximization (OSEM) algorithm [22] using a Hermes workstation (Nuclear Diagnostics, UK). Attenuation correction and collimator blurring correction was performed during the iterative reconstruction. No filtering was applied after reconstruction. For each phantom measurement, one of 2 attenuation correction options and 3 collimator blurring correction options were performed resulting in 6 different combinations of reconstructions per acquisition. The 2 attenuation correction options were (1) uniform AC using Chang's method (*μ* = 0.11/cm), and (2) nonuniform AC using the low-dose CT study. The 3 collimator blurring correction options were (1) no correction, (2) correction using a constant point spread function, (3) correction using a point spread function which varied as a function of distance from the collimator.

All reconstructions were performed using 30 subsets and 10 iterations. This was chosen to ensure convergence [23] of the mean activity in a region and was based on the analysis of a single patient study using ^123^I-fluoropropyl-carbomethoxy-3*β*-(4-iodophenyltropane) (FP-CIT). On the measured attenuation maps, the phantom chambers were noted to have an attenuation coefficient of about 0.15/cm. This value is consistent with what would be expected for a 0.1 M sodium iodide solution [24]. In order to determine the effect of the *μ* value used during the attenuation correction on SUR, we reconstructed a single phantom study with *μ* values varying from 0 to 0.5/cm. Specifically for *μ* values of 0.10/cm to 0.15/cm, the SUR values varied from 2.68 to 2.76 leading to a maximum variation of 3%. Uniform attenuation correction was therefore performed according to EANM guidelines using *μ* = 0.11/cm [21, 25]. Placement of the scalp ellipse was complicated in the phantom studies by the absence of scalp activity. Using an attenuation map it was established that the average scalp thickness on the phantom was 11.8 mm (4 pixels). The position of the scalp was therefore approximated by placing an ellipse over the edge of the brain and expanding it radially by 11.8 mm (4 pixels).

Depth-dependent collimator blurring was performed using a linear relationship between distance and the full width at half maximum (FWHM) of the point spread function. We used two different methods in this study: method 1 was based on a measurement (intercept = 3.5 mm FWHM, slope = 0.033 mm/mm) while method 2 (intercept = 5.3 mm FWHM, slope = 0.075 mm/mm) was obtained by determining the values which corresponded to an optimal ratio of measured SUR to true SUR for a single independent phantom study. Nondepth-dependent correction for collimator blurring was performed using a constant point spread function of 9.4 mm FWHM or 16.3 mm FWHM. The first value corresponds to the default value in the Hermes software in our institution, while the latter was again obtained by the optimization procedure described above for the depth dependent method. 

Each of the reconstructed images was smoothed using a Gaussian kernel varying from 1 to 16 mm FWHM in 1 mm increments.

### 2.4. Analysis

**Figure 1:**
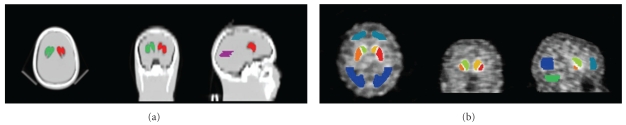
Striatal (red, green) and occipital (purple) volumes of interest used for the phantom study defined using the CT image of the phantom acquired with water in the background chamber and air in the striatal chambers (a). For the patient study using the Hermes Brass software striatal (light green, orange, red, yellow) and occipital (dark blue) volumes were used (b). Frontal (light blue) and cerebellar (green) volumes were not used for this study.

A phantom CT study was performed with the main brain volume filled with water, while the striatal volumes contained air, which will be referred to as the phantom-air study. The CT image derived from the phantom-air study, and all reconstructed SPECT images and CT-based attenuation maps from the 8 phantom SPECT-CT studies were converted from interfile to ANALYZE format using MRIcro software [26]. The reconstructed SPECT images and CT based attenuation maps were all coregistered to the same space as the phantom-air study using a mutual information algorithm with Statistical Parametric Mapping software (SPM version 2, http://www.fil.ion.ucl.ac.uk/spm/, Wellcome Department of Cognitive Neurology, UK). Using MRIcro and the phantom-air CT image, 3-dimensional VOIs were drawn for the left striatum, right striatum, and a background volume located posteriorly in the main brain volume (Figure 1(a)). In each voxel of the two striatal VOIs, the specific uptake ratio (SUR) was calculated using formula (1) with the background activity being equal to the mean activity within the background VOI and the “striatal activity” being the activity in the voxel. For each VOI, a mean SUR_mean_ and standard deviation SUR_SD_ was determined. These values were determined for each VOI (*v*), for each phantom measurement (*p*), for each type of reconstruction (*r*), and for each smoothing kernel (*s*).

Since we would like to compare the different reconstructions with a variable smoothing kernel, the average recovery (AR) and root mean squared coefficient of variance (RMSCOV) was then determined across all phantoms and VOIs using the following formulae:

(2) $$\begin{array}{c} \text{AR}\left(r,s\right)=\frac{100}{n_{p}n_{v}}\sum_{v,p}\frac{\text{SU}{\text{R}}_{\text{mean}}\left(v,p,r,s\right)}{\text{REF}\left(v,p\right)} \end{array},$$

(3) $$\begin{array}{c} \text{RMSCOV}\left(r,s\right)=100\cdot \sqrt{\frac{1}{n_{p}n_{v}}\sum_{v,p}{\left(\frac{\text{SU}{\text{R}}_{\text{SD}}\left(v,p,r,s\right)}{\text{SU}{\text{R}}_{\text{mean}}\left(v,p,r,s\right)}\right)}^{2}}, \end{array}$$

where REF(*v*, *p*) is the reference SUR value for a VOI *v* and phantom measurement *p* as determined by the well counter measurements.

*n*_*p*_ and *n*_*v*_ are the number of phantom measurements and the number of VOI's, respectively.

These two values (both expressed in %) represent a measure for “bias” (less bias corresponds to higher AR) and “noise” (RMSCOV), respectively. The RMSCOV was plotted against the average recovery (AR), with different amounts of smoothing producing a “bias-noise” curve for each type of reconstruction.

In addition, using a similar methodology to Koch et al. [14], the measured SUR determined from the SPECT measurement and the true SUR determined from the samples taken from the phantom chambers, were analysed using scatter plots and compared by simple linear regression analysis.

### 2.5. Patient Measurements

Fourteen patient studies were performed on adult subjects with a primary diagnosis of generalised social anxiety disorder (SAD) recruited from the Anxiety Disorders Clinic of our tertiary hospital. All patients were interviewed with the Structured Clinical Interview for the Diagnosis of Axis-I Disorders to ascertain the diagnosis according to diagnostic and statistical manual of mental disorders (DSM IV) criteria [27]. Patients with other primary psychiatric disorders, significant medical illness, or a neurological condition were excluded. No patients were receiving psychotropic medication at the time of the study. SPECT-CT scanning was performed after approval was received from the Ethical Review Board of our institution.

### 2.6. SPECT-CT Imaging and Reconstruction

SPECT-CT imaging was performed 3 hours after the administration of a mean (standard deviation (SD)) dose of 134 (26) MBq of [I-123]-(FP-CIT). SPECT-CT imaging was identical in every respect to that performed for the phantom study, with the exceptions of the rotational radius and the acquisition time. The rotational radius was chosen so that the collimators were as close as possible to the patient's head, resulting in a variation of 14.2–16.9 cm. The time for each projection was 20 seconds in 5 patients, 25 seconds in 8 patients, and 30 seconds in 1 patient. Reconstruction was identical to that performed for the phantom data. Acquisition was done according to 2010 EANM guidelines [21] except in 3 patients (acquired counts at least 2.5 M instead of 3 M).

### 2.7. Analysis

All SPECT images were transformed to the same space by fitting to a FP-CIT template using BRASS software (Hermes Medical Solutions, Sweden) [28, 29]. This was done by applying a filter (Butterworth, order 5, cut off 0.9 cm^−1^) to the unfiltered SPECT image of each patient obtained using nonuniform attenuation correction and correction for collimator blurring using a variable point spread function. This filtered image was then fitted to the FP-CIT template using BRASS. The transform obtained was then applied to each of the unfiltered images for the 6 different attenuation corrections and collimator blurring options for that patient.

Quantification was performed with VOIs used by the Brass software for the left and right caudate, left and right putamen, and a posterior background VOI (Figure 1(b)). The VOIs and the transformed SPECT images were converted from interfile to ANALYZE format using MRIcro software. For each of the 4 striatal VOIs and each of the 14 patients, a mean specific uptake ratio (SUR_mean_) and its standard deviation (SUR_SD_) were determined similarly to the phantom study. The RMSCOV was again calculated using (3) above for each reconstruction and smoothing kernel. For the patient studies a true measure of striatal uptake was not available. Therefore, for each reconstruction *r* and smoothing *s*, the average SUR (aSUR) was determined using the following formula:

(4) $$\text{aSUR}\left(r,s\right)=\frac{1}{n_{p}n_{v}}\sum_{v,p}\text{SU}{\text{R}}_{\text{mean}}\left(v,p,r,s\right),$$

where *n*_*p*_ and *n*_*v*_ are the number of patients and the number of VOIs respectively.

It has been shown previously that the measured specific uptake ratio SUR is proportional to the true SUR [14]. It can therefore be expected that aSUR is proportional to AR, making it a useful surrogate measure when true values are not available. Therefore, we used aSUR as an alternative to the average recovery AR.

Similar to the phantom studies, RMSCOV was plotted against the aSUR, with different amounts of smoothing producing a surrogate “bias-noise” curve for each combination of attenuation and collimator blurring correction.

### 2.8. Statistical Analysis

**Figure 2:**
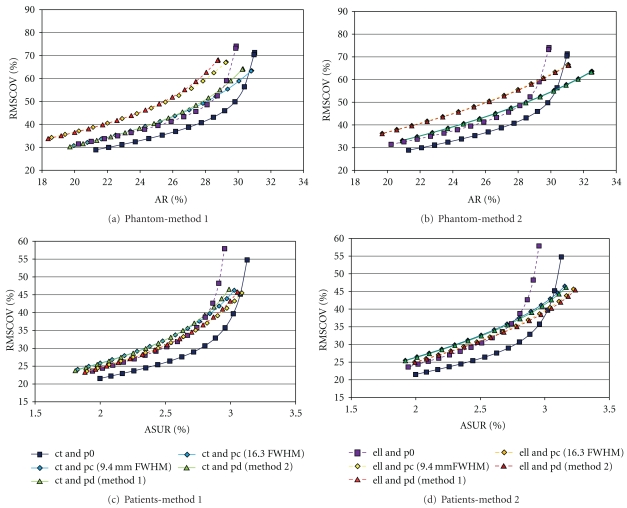
“Bias-noise” curves: plot of root mean squared coefficient of variation (RMSCOV) versus average recovery (AR) for phantom data (a and b) or versus the average specific uptake ratio (aSUR) for patient data (c and d). (a) give the results when using no correction for collimator blurring, the default value (9.4 mm FWHM) for the constant collimator blurring correction, and the measured depth dependent collimator blurring correction (method 1, see text). (d) give the results when using the optimized values for the constant collimator blurring correction or the depth dependent collimator blurring correction (method 2, see text). In the latter figures, we added the case when no correction for collimator blurring is performed to help in the comparison with the top figures. (abbreviations: ct: nonuniform attenuation correction using a CT-based attenuation map; ell: uniform attenuation correction using an ellipse approximating the scalp; p0, pc, pd: no, constant, and depth dependent correction for collimator blurring, resp.; FWHM: full width at half maximum).

Evaluation of the curves requires that comparison be performed for similar levels of noise, or similar levels of bias, or a combination of the two. For our analysis, we chose to minimize bias (maximal AR or aSUR), while still having similar noise levels. This was done by first identifying the point of minimal bias (AR/ASUR) across all reconstructions. The AR/ASUR at that point was then compared to the AR/ASUR for other reconstructions at similar noise levels. This was done by first identifying the point of minimum bias (AR/ASUR) across all reconstructions. The AR/ASUR at that point was then compared to the AR/ASUR for other reconstructions at similar noise levels. This approach was used as a simple comparison of points of minimal AR/ASUR for each reconstruction would be difficult to interpret given the large differences in noise (RMSCOV) for the different techniques. Comparison was performed using a 2-way analysis of variance (ANOVA) with repeated measures (factor 1: uniform or nonuniform attenuation correction, factor 2: no, constant, or depth dependent collimator blurring correction). This resulted in unsmoothed collimator blurring reconstructions being compared to reconstructions without collimator blurring with a similar RMSCOV (Figures 2(a) and 2(b)). For the phantom study, the measurements in each of the two striatal compartments were taken separately in the 2-way ANOVA using repeated measures, leading to 14 measurements per method. For the patient study, the measurements of each of the 4 striatal VOIs were taken separately leading to 56 measurements per method. Findings were considered significant for *P* < .05 and reported as a trend for *P* < .1.

## 3. Results

### 3.1. Phantom Measurements

Data was acquired using 7 phantom SPECT-CT studies with 14 different specific uptake ratios of I-123. True specific uptake ratio values were varied from 2.08 to 14.70. Using unsmoothed nonuniform attenuation and depth dependent collimator blurring corrections, measured SUR values varied from 0.58 to 5.05. Total counts for the studies varied from 3.2 to 3.8 million. There was a close linear correlation (all with *R* ^2^ > 0.96) between the measured and true SUR values for all reconstruction techniques with higher values for the true SUR values, consistent with findings described previously [14]. 

The “bias-noise” (AR-RMSCOV) curves are shown in Figures 2(a) and 2(b). The curves obtained show a higher average recovery (AR) and lower RMSCOV using nonuniform attenuation correction compared to uniform attenuation correction. This was observed for images obtained both with and without corrections for collimator blurring.

Comparing the different methods (Figure 2(a)) using the default value for the constant collimator blurring and the measured depth dependent linear relation between psf and distance (method 1, see Methods), we found a significant main effect of the attenuation correction method (*F*(1,13) = 5.67, *P* = .033) with higher AR values when applying a CT based attenuation correction. No main effect of the method of correcting for collimator blurring was found. A significant interaction effect between the correction for attenuation and the correction for collimator blurring was observed (*F*(2,26) = 3.45, *P* = .047). Comparing the different methods (Figure 2(b)) using the optimized values for constant and depth dependent collimator blurring correction (method 2, see methods), we found a similar main effect of the attenuation correction method (*F*(1,13) = 4.92, *P* = .045) but in this case we also found a significant main effect of the method for correcting for collimator blurring (*F*(2,26) = 12.47, *P* < .001) with increased values of AR for both the constant and the depth-dependent correction compared to no correction. The interaction effect showed a trend (*F*(2,26) = 3.24, *P* = .056). 

Unsmoothed reconstructions performed with collimator blurring corrections (method 2) had a higher average recovery (AR), and a lower noise (RMSCOV) compared to reconstructions without collimator blurring corrections (Table 1). However it is interesting to note that smoothing of the images did not have the same effect on images obtained with and without corrections for collimator blurring. Smoothing of reconstructions obtained using collimator blurring corrections appears to be of little value as the average recovery AR rapidly decreases for a relatively modest decrease in RMSCOV. With smoothing using a Gaussian kernel of larger than about 5 mm FWHM, the AR-RMSCOV curves of these reconstructions cross over the curves for reconstructions without collimator blurring corrections (Figure 2(b)), indicating that they then perform less well. Conversely, smoothing of reconstructions performed without collimator blurring initially results in a marked improvement in the RMSCOV with a relatively modest loss in average recovery AR.

### 3.2. Patient Measurements

Fourteen patient studies were performed. Using unsmoothed nonuniform attenuation correction and depth dependant collimator blurring corrections, measured specific uptake ratio (SUR) values varied from 1.49 to 5.43. Total counts for the studies varied from 2.5 to 6.4 million. The aSUR-RMSCOV curves are shown in Figures 2(c) and 2(d). Results obtained from patient studies are similar to those obtained from phantom data however some key differences were noted.

The curves obtained again show a higher aSUR and lower RMSCOV using nonuniform attenuation correction compared to uniform attenuation correction, for images obtained without corrections for collimator blurring. However a key difference was noted with the patient curves when compared to those obtained with phantom data. Collimator blurring corrected reconstructions obtained with nonuniform attenuation correction resulted in lower aSUR values than those with uniform attenuation correction. The opposite was true for the phantom data.

Comparing (at a RMSCOV level similar to unsmoothed reconstructions with collimator blurring correction) the different methods (Figure 2(c)) using the default value for the constant collimator blurring and the measured depth dependent linear relation between psf and distance (method 1, see Methods), we found a significant main effect for the type of correction for collimator blurring (*F*(2,110) = 3.44, *P* = .036) as well as a significant interaction effect between the method for attenuation correction and the method for correction of collimator blurring (*F*(2,110) = 23.84, *P* < .001). No significant main effect of the attenuation correction method was found. 

The same results were found when comparing (at a RMSCOV level similar to unsmoothed reconstructions with collimator blurring correction) the different methods (Figure 2(d)) using the optimized values for constant and depth dependent collimator blurring correction (method 2, see methods): a significant main effect of the method for collimator blurring (*F*(2,110) = 23.42, *P* < .001) as well as a significant interaction effect (*F*(2,110) = 26.23, *P* < .001). 

For reconstructions with uniform attenuation correction, correcting for collimator blurring (either constant or depth dependent) achieved significantly higher aSUR values than not correcting for collimator blurring. This however was not the case for reconstructions with nonuniform attenuation correction, with the aSUR values not being significantly higher. From another perspective, unlike the phantom data, for this patient population given similar RMSCOV values (i.e., at similar noise levels), reconstructions with uniform attenuation correction combined with correction for collimator blurring achieve the highest average specific uptake ratio (aSUR).

## 4. Discussion

In this study, the effects of (1) nonuniform attenuation correction and (2) correction for collimator blurring were determined for striatal SPECT imaging. It was found that nonuniform attenuation correction resulted in improved quantification of striatal SPECT images. When using an additional correction for collimator blurring, the bias was lowest (i.e., the highest average recovery AR). This however was not seen with the patient studies, with the combination of a uniform attenuation correction and a correction for collimator blurring leading to the lowest bias (i.e., the highest average SUR).

The different techniques used in this study resulted in images of differing smoothness, even after applying the same postsmoothing, complicating the comparison of the reconstruction methods. Without taking this into account it is possible that one technique may appear superior when this is in fact not the case. The creation of “bias-noise” curves (in our study represented by AR-RMSCOV and aSUR-RMSCOV curves) assisted with a comparison of these techniques independent of this potentially important confound. A noise (RMSCOV) level similar to that of unsmoothed collimator blurring corrected images was chosen to compare the methods as this was regarded as a level that is appropriate for reading of the scans visually. The “bias-noise” curves enabled the selection of appropriate points on curves for reconstructions without collimator blurring for comparison. In addition the curves made it possible to observe the differing effects of smoothing on images reconstructed in different ways. In the case of images generated using collimator blurring corrections, further smoothing appears to be of little value resulting in a rapid decline in average recovery AR or average specific uptake ratio aSUR, with a modest decrease in noise. Conversely, a degree of smoothing or filtering is essential for images reconstructed without collimator blurring, resulting in a marked decrease in noise with little loss in the average recovery AR or specific uptake ratio aSUR. 

Nonuniform attenuation correction alone as opposed to uniform attenuation correction alone resulted in SPECT images with specific uptake ratio values that were closer to the true values known from phantom data. These results concur with previous work suggesting some benefit in the use of nonuniform attenuation correction for brain SPECT [10–12].

The use of corrections for collimator blurring during reconstruction resulted in a marked increase in average recovery (AR) and average specific uptake ratio (aSUR) values. In this study, striatal SPECT data obtained using both an anthropomorphic phantom, and patient studies provide support for the results of previous studies using simulated data [6, 7]. This was true for corrections using both a constant and a depth-dependant PSF. Although a depth-dependant PSF resulted in a higher average specific uptake ratio aSUR value, compared to a constant PSF, the differences were very small.

**Figure 3:**
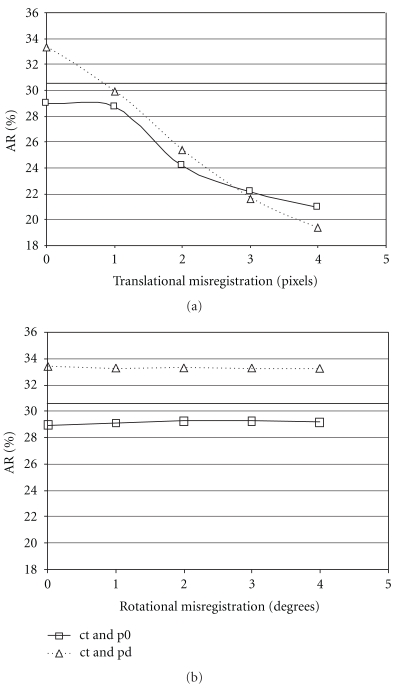
Plot of average recovery (AR) for a single phantom study (using unsmoothed data) as a function of translational SPECT-CT mis-registration (perpendicular to the tomographic axis of rotation, 1 pixel = 2.95 mm) or rotational SPECT mis-registration (around the axis of the tomographic axis of rotation), (abbreviations: ct: nonuniform attenuation correction using a CT-based attenuation map; p0, pd: no, and depth dependent correction for collimator blurring, resp., based on the optimized method). The solid line gives the AR value calculated using uniform attenuation correction and depth dependent correction for collimator blurring.

Perhaps the most interesting findings of this study were the results obtained when nonuniform attenuation correction and collimator blurring corrections were used together. Intuitively, one would expect that this combination would result in a further increase in the average recovery and specific uptake ratio. This was indeed the case for the values obtained using the phantom data. The use of both correction methods resulted in an average recovery that was increased compared to the case when nonuniform attenuation correction alone was applied. This was also the case when a uniform attenuation correction was applied in combination with a collimator blurring correction. However, the findings for the patient data revealed a different result. For the patient studies, the highest average specific uptake ratio aSUR was achieved using collimator blurring corrections in combination with a uniform attenuation correction. The reason for this finding is not immediately clear; however the combination of patient data, nonuniform attenuation and collimator blurring corrections unexpectedly leads to a suboptimal result. It can be speculated that patient studies are likely to have small misregistrations between the SPECT and CT studies due to patient movement between the acquisitions, something that should not occur with a phantom study. These small mis-registrations may not have a major impact on reconstructions with nonuniform attenuation correction for the striatal regions but reconstructions with collimator blurring corrections may be more sensitive to them. Based on a visual assessment, there were no marked mis-registrations of the patient data used in this study, but it is possible, however, that there may be subtle differences, not seen with visual inspection. To further test this explanation, a single phantom study was taken and deliberate SPECT-CT translational and rotational mis-registrations were introduced. The mis-registered data were then reconstructed using uniform attenuation correction combined with a depth dependent collimator blurring correction, nonuniform attenuation correction alone, and nonuniform attenuation correction combined with a correction for collimator blurring. Rotational mis-registration up to 4 degrees was found to have little effect on striatal AR, but a translational mis-registration of 3–6 mm (1-2 pixels) gave a result similar to that obtained for the patient data above, that is, the highest recovery was obtained for reconstruction with a uniform attenuation correction and combined with a collimator blurring correction (Figure 3). 

This study shows that care must be taken with extrapolating the optimal reconstruction technique derived from phantom data to real patient data. In practice, we do have to deal with small deviations from the perfect situation and a method sensitive to small errors may not be optimal. This might be the case when using a CT-based attenuation correction combined with a correction for collimator blurring.

The AR for the phantom study varied from about 28 to 33%, which is relatively low compared to data from simulation studies. However, this is comparable with published work with anthropomorphic phantom studies using a similar dual headed gamma camera-collimator combination [14].

Based on the results of our study, we can select the optimal reconstruction technique depending on the choice of the noise level, the level of bias, or a combination. In Tygerberg Hospital, we aim for images with low bias and an acceptable level of noise. Therefore, we are currently using a uniform attenuation correction combined with a depth dependent correction for collimator blurring using the optimized method.

## 5. Conclusion

This study suggests that particularly when quantification is required, an optimal brain SPECT reconstruction (in terms of the lowest bias) in patients would include a correction for collimator blurring and uniform attenuation correction.

## Figures and Tables

**Table 1:** Average recovery (AR) and root mean squared coefficient of variation (RMSCOV) values for unsmoothed reconstructions, (abbreviations: p0, pc, pd: no, constant, and depth dependent correction for collimator blurring resp.).

	Collimator blurring	AR	RMSCOV
CT	p0	31.0	71.3
Based	pc (method 1)	30.8	63.4
Attenuation	pc (method 2)	32.5	63.7
Correction	pd (method 1)	30.3	64.3
	pd (method 2)	32.5	63.4

Uniform	p0	29.9	74.1
Based	pc (method 1)	29.3	67.1
Attenuation	pc (method 2)	31.1	66.6
Correction	pd (method 1)	28.8	68.1
	pd (method 2)	31.1	66.4
